# Employing knowledge translation interventions to increase the use of therapeutic hypothermia post arrest: the SPARC Network Trial

**DOI:** 10.1186/cc9728

**Published:** 2011-03-11

**Authors:** LJ Morrison, P Dorian, KN Dainty, S Brooks, K Thorpe, C Zhan, D Scales

**Affiliations:** 1 University of Toronto, Canada

## Introduction

Current guidelines recommend early institution of therapeutic hypothermia (TH) in survivors of out-of-hospital cardiac arrest (OHCA). However, recent surveys show that TH is delivered inconsistently, incompletely, and with undue delay. Targeted knowledge translation (KT) strategies may increase the proportion of OHCA patients receiving TH

## Methods

We conducted a stepped-wedge cluster randomized trial to evaluate the effectiveness of a multi-faceted KT strategy for increasing TH use in a network of 37 hospitals. After a baseline period of 1 year, four wedges of six hospitals were randomized to receive 1 year of passive KT followed by 4 months of active KT. Passive KT included a generic protocol and order set; active KT included network events, performance feedback and ongoing nurse educator support. The primary outcome was the rate of successful TH, defined as a temperature of 32 to 34°C within 6 hours of emergency department (ED) arrival.

## Results

**Figure 1:**
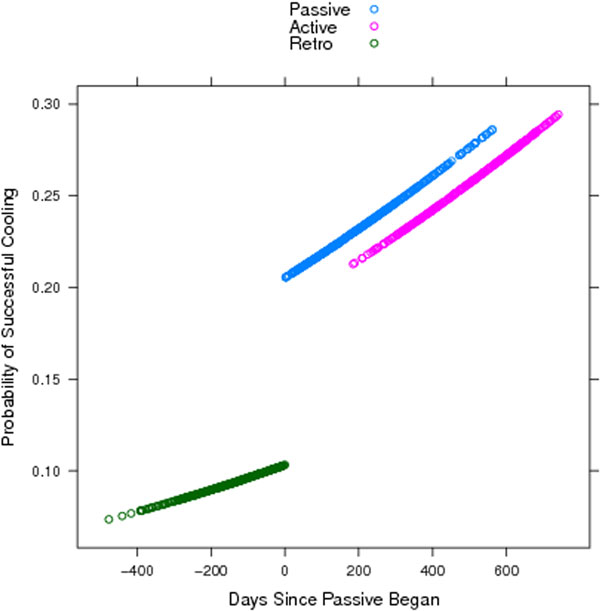
**Successful TH across KT interventions**.

During the study 4,742 OHCA patients were transported to hospital and 1,063 (22%) were eligible for TH. Overall, both KT interventions were effective at increasing the rate of successful TH (Figure 1), and passive KT led to marked improvements over baseline (96/395 vs. 30/320 patients; OR = 2.24, 95% CI = 1.54 to 3.26; *P *< 0.05). Active KT did not improve the primary outcome compared with passive KT (86 of 348 patients with active KT; OR = 0.94, 95% CI = 0.70 to 1.28; *P *= 0.70); however, it did significantly increase rates of initiating TH in the ED (*P *= 0.04). Inappropriate TH remained rare (5 to 6% of patients) during both KT phases.

## Conclusions

A multifaceted KT intervention markedly improved rates of TH in a large network of hospitals. Simple passive KT strategies were highly effective in increasing TH rates, whereas more active KT improved the use of TH in the ED.

